# Determinants of Physical Health Self-Management Behaviours in Adults With Serious Mental Illness: A Systematic Review

**DOI:** 10.3389/fpsyt.2021.723962

**Published:** 2021-08-18

**Authors:** Peter A. Coventry, Ben Young, Abisola Balogun-Katang, Johanna Taylor, Jennifer V. E. Brown, Charlotte Kitchen, Ian Kellar, Emily Peckham, Sue Bellass, Judy Wright, Sarah Alderson, Jennie Lister, Richard I. G. Holt, Patrick Doherty, Claire Carswell, Catherine Hewitt, Rowena Jacobs, David Osborn, Jan Boehnke, Najma Siddiqi

**Affiliations:** ^1^Department of Health Sciences, University of York, York, United Kingdom; ^2^Institute of Health and Wellbeing, University of Glasgow, Glasgow, United Kingdom; ^3^Hull York Medical School, University of York, York, United Kingdom; ^4^School of Psychology, University of Leeds, Leeds, United Kingdom; ^5^School of Medicine, University of Leeds, Leeds, United Kingdom; ^6^Human Development and Health, Faculty of Medicine, University of Southampton, Southampton, United Kingdom; ^7^University Hospital Southampton National Health Service Foundation Trust, Southampton, United Kingdom; ^8^Centre for Health Economics, University of York, York, United Kingdom; ^9^Division of Psychiatry, University College London, London, United Kingdom; ^10^School of Health Sciences, University of Dundee, Dundee, United Kingdom

**Keywords:** self-management, behaviour change, serious mental illness, determinant, theory

## Abstract

Behavioural interventions can support the adoption of healthier lifestyles and improve physical health outcomes, but it is unclear what factors might drive success of such interventions in people with serious mental illness (SMI). We systematically identified and reviewed evidence of the association between determinants of physical health self-management behaviours in adults with SMI. Data about American Association of Diabetes Educator's Self-Care Behaviours (AADE-7) were mapped against the novel Mechanisms of Action (MoA) framework. Twenty-eight studies were included in the review, reporting evidence on 104 determinant-behaviour links. Beliefs about capabilities and beliefs about consequences were the most important determinants of behaviour, especially for being physically active and healthy eating. There was some evidence that emotion and environmental context and resources played a role in determining reducing risks, being active, and taking medications. We found very limited evidence associated with problem solving, and no study assessed links between MoAs and healthy coping. Although the review predominantly identified evidence about associations from cross-sectional studies that lacked validated and objective measures of self-management behaviours, these findings can facilitate the identification of behaviour change techniques with hypothesised links to determinants to support self-management in people with SMI.

**Systematic Review Registration:** PROSPERO, registration CRD42018099553.

## Introduction

Adults with serious mental illness (SMI), such as schizophrenia or bipolar disorder, experience considerable inequalities in health outcomes compared with the general adult population. Life expectancy for individuals with SMI is 10–20 years shorter and the mortality rate 3.7 times higher than in the general population (1–4). Furthermore, this mortality gap is widening (5). It is estimated that two thirds of these deaths are attributable to preventable long-term physical conditions such as cardiovascular disease, respiratory disease, diabetes and hypertension (1, 6). There is at least a 2-fold greater prevalence of obesity, diabetes, and cardiovascular disease in adults with SMI compared with the general adult population (6–8).

Supported self-management is critical to prevention and improving outcomes of long-term physical conditions and there is robust evidence that behavioural interventions can effectively support people in the general population to self-manage their health (9). Self-management refers to activities undertaken by individuals, typically to mitigate the effects of a long-term condition and maximise quality of life. Self-management of physical health comprises a range of health behaviours that include diet, physical activity, smoking abstinence, self-monitoring, and seeking appropriate professional help.

The evidence for behavioural interventions to support self-management in people with SMI is limited. There is some evidence that prescribed and directly administered exercise interventions that include up to 90 min a week of moderate-to-vigorous exercise can improve physical fitness and cardiometabolic risk as well as reduce psychiatric symptoms in people with schizophrenia (10). However, there is limited evidence that behavioural interventions positively affect physical activity in people with SMI. Findings from a systematic review of 32 studies of behavioural interventions to promote physical activity and reduce sedentary behaviours in people with schizophrenia were inconsistent and based on low quality evidence from controlled and uncontrolled trials (11). The evidence that behavioural approaches that include lifestyle interventions to support dietary change and physical activity to reduce weight in people with SMI is similarly equivocal. Naslund et al. reported small but significant treatment effects across 17 experimental and quasi-experimental studies of lifestyle weight loss interventions in overweight and obese people with SMI (12). However, findings from a Danish trial that tested an intensive lifestyle coaching intervention plus care coordination for people with schizophrenia-spectrum disorder and obesity which failed to show any positive results for 10-year cardiovascular risk factors or weight reduction (13). Efforts to target multiple cardiovascular risk factors using manualised and supported behavioural interventions in people with SMI have also proven ineffective (14). The STEPWISE trial tested the effectiveness of a group-based intervention, with 1:1 fortnightly telephone support, to identify and encourage ways to achieve dietary and physical activity goals in people with schizophrenia. The intervention was based on self-regulation and self-efficacy theories and a relapse prevention model, and was co-designed in partnership with people with lived experience of SMI, mental health professionals and behaviour change experts. However, weight reduction did not differ between intervention and control groups, and other key indicators of self-management, such as physical activity, remained unchanged (15).

Living with SMI may pose significant barriers to engaging in self-management of physical health. Individuals with SMI spend less time being physically active (16), are less likely to eat a healthy diet (17), and more likely to smoke than other people (18). There are a number of potential reasons for this, including how psychiatric symptoms can inhibit self-management behaviours. People with SMI experience deficits that are commonly referred to as negative symptoms; these include avolition, psychomotor retardation, blunted affect, alogia and anhedonia (19). People with SMI also experience positive symptoms of psychosis, including delusions and hallucinations. Negative symptoms have been shown to predict poorer cardiorespiratory fitness, larger waist circumference, higher HbA1c, and lower high-density lipoprotein in overweight people with schizophrenia (20). Both negative and positive symptoms can influence a person's ability to engage in health behaviours, either by directly impacting their motivation and their ability to understand the importance of these behaviours, or through triggering the use of unhealthy behaviours to cope with symptomatic episodes (21). The presence of psychiatric symptoms has been shown to overshadow diabetes self-management in people with SMI (22). Additionally, antipsychotic medications are commonly used to manage psychosis and are associated with increased risk of obesity, excessive weight gain and metabolic derangement (23, 24). Antipsychotics can also make self-management more difficult through unwanted side-effects, such as increased appetite and sedation (25, 26).

Over and above individual level factors, social and community level factors also underscore health inequalities experienced by people with SMI. People with SMI are more likely to experience higher levels of deprivation than the general population (27) and SMI increases the odds of living in poverty (28). Indeed, inequalities in mental health outcomes can in part be explained by neighbourhood and area of residence (29) and recent spatial analyses at small area level across England has shown higher prevalence of SMI in socially fragmented and socially deprived areas (30).

### Intervention Development Methods and Theoretical Framework

To maximise the chance that behavioural interventions to support physical health self-management in people with SMI are effective and sustainable, an approach that draws on the science of behaviour change is needed. Intervention development in such an approach proceeds by the description of behavioural targets that drive risk factors, identification of mechanisms of action through which behaviour change might occur, followed by the identification of specific techniques that might alter the target, and the formulation of process measures that can measure the extent to which the intervention was successful (31). Our approach draws on a phased based approach underpinned by the Medical research Council Framework for developing and evaluating complex interventions (32). In the context of the science of behaviour change our work methodologically maps to the Behaviour Change Wheel (33) and the Obesity-Related Behavioural Interventions Trials or ORBIT model (34). These approaches within the science of behaviour change are well-suited to an emphasis on the early phases of intervention development, starting with the identification of hypothesised pathways that might mediate behaviour change and a clinical outcome, and the refinement and preliminary testing of an intervention in readiness for definitive phase III testing.

In order to design appropriate and effective supported physical health self-management interventions for people with SMI, it is essential to first identify modifiable determinants of behaviour change in this population. There is currently effort underway to develop ontologies as a means of building toward unifying different health psychological theories that speak to the range of influences upon behaviour (35). Contributing toward this, behavioural science has developed methods to systematically describe potentially active intervention components to support development, implementation, and evaluation of interventions (36). To facilitate intervention development, there is a need to identify and map evidence about the relationship between determinants and behaviours in a way that can guide the selection of appropriate intervention components. Interventions that address modifiable determinants might be more effective in changing behaviour. The Theoretical Domains Framework (TDF) contains 14 domains based on an integration of behavioural theories that relate to individual processes and characteristics of the physical and social environment that may act as determinants of (health) behaviour (37). The framework is itself an elaboration of the Capability—Opportunity—Motivation—Behaviour (COM-B) model that underpins the widely used Behaviour Change Wheel intervention development framework (33). *Capability* relates to a person's psychological and physical capacity to undertake a behaviour, including know-how and skills to do so. *Opportunity* concerns all the available social and physical factors within a person's environment that make the behaviour possible, while *Motivation* is specified as both reflective processes associated with planning and automatic processes associated with emotional responses, reactions, and impulses. The COM-B model proposes that capability and opportunity can influence motivation, to bring about behaviour through both direct and indirect paths. There is emerging evidence that COM-B outperforms other more established models of behaviour such as the theory of planned behaviour, theory of reasoned action, and the health belief model, in explaining the variance in delivery of opportunistic behaviour change interventions and the variance in time spent delivering interventions (38). Because the COM-B model forms the hub of the behaviour change wheel it can be used to identify potentially relevant intervention functions that could be deployed to target determinants of behaviours.

More recently, Michie et al. have combined the TDF components with 12 other mechanisms which did not overlap with the TDF and were identified in a literature review of 83 behaviour change theories. This process resulted in 26 Mechanisms of Action (MoAs) with expert rated links to 56 frequently used behaviour change techniques (39, 40). The findings from the literature review and expert consensus exercise were then triangulated to systematically produce evidence of 92 hypothesised behaviour change techniques (BCT)-MoA links with the potential to be targeted by interventions, along with evidence about where links do not exist or are inconclusive (41). This evidence has been distilled into an online tool known as the Theory and Techniques Tool which offers a comprehensive and efficient system to identify intervention techniques that are purported to operate through theoretically informed MoAs (41). Given the multiple theories that offer frameworks with which to identify processes by which behaviour change interventions operate (42), synthesis of theoretical approaches is required to avoid narrowing the available evidence (43). We applied the MoA framework to integrate evidence that spans a variation in populations, context, and behaviour (44). To be useful as an evidence synthesis tool for intervention development, it is necessary that that any theoretical framework or theory for identifying mechanisms of action also provides a taxonomy of behaviour change intervention techniques with which to support integration of evidence for both the mechanisms and the technique that targeted it. With a view to informing the identification and potential adaptation of behaviour change interventions to support self-management of physical health in people with SMI, we therefore aimed to systematically review the literature to identify the MoAs that determine self-management behaviours in adults with SMI, including those who have co-morbid long-term physical health conditions.

## Methods

### Protocol and Registration

This systematic review forms the first phase of work of the DIAMONDS research programme that is dedicated to developing, piloting, and then definitively testing a supported self-management intervention based on evidence based behaviour change techniques for people with SMI and diabetes (45). Our review maps to Phase 1a of the ORBIT model for developing behavioural interventions. The protocol was prospectively registered with PROSPERO, registration CRD42018099553. Amendments to the protocol are summarised in Table 1. The review addressed two questions:

- What are the determinants of self-management behaviours that underpin physical health in adults with SMI?
- How do these determinants differ for people with SMI who have co-morbid long-term physical health conditions?

**Table 1:** Amendments to protocol.

**Section**	**Original protocol**	**Revised protocol**	**Rationale**
Review question	What are the determinants of self-management in adults with SMI?	What are the determinants of physical health self-management in adults with SMI?	The number of included studies exceeded expectations and physical health self-management was prioritised to address the SMI mortality gap
Inclusion criteria	Inclusion of qualitative study designs	Inclusion of quantitative research only	Included studies were grouped by quantitative/qualitative design and a separate synthesis of qualitative evidence undertaken for pragmatic reasons, i.e., to ensure the work was feasible according to available resources
Inclusion criteria	Exclusion of non-English language reports	No exclusions on language	To maximise retrieval of all relevant studies and utilise local translation services
Inclusion criteria	No restrictions on setting	Exclusion of studies of inpatients	The inpatient setting is likely to involve different determinants of self-management
Quality appraisal	Assessment of study quality using a framework developed for mixed methods reviews	Use of the NICE quality appraisal checklist for quantitative studies reporting correlations and associations	Use of a tool appropriate for the design of included studies
Data extraction	Extraction of a random 20% sample of data independently by a second reviewer to identify any discrepancies	A second reviewer extracted data independently from 4 studies which was checked by the first reviewer to identify any discrepancies	To make use of data that had already been extracted from studies of individuals with a long term physical condition that were prioritised to inform the intervention to be developed as part of the wider project
Data synthesis	Synthesis of determinates of self-management using the Capabilities, Opportunities, Motivations and Behaviours (COM-B) system	Synthesis of determinates of self-management using the Mechanisms of Action (MoAs) framework	The Theory and Techniques tool was published since the review protocol and provides evidence-based links between the MoAs and behaviour change techniques, therefore the MoAs were deemed more useful than the COM-B in informing intervention content

### Eligibility Criteria

Studies were eligible if they reported determinants of self-management of physical health in adults with SMI. In this review physical health relates to a dynamic state related to a person's ability to self-manage and restore functional capacity and well-being (46). Determinants of self-management were first identified using the COM-B model (capability, opportunity, motivation, and behaviour) (33). Self-management behaviours were defined as “all the actions undertaken by people to recognise, treat and manage their own healthcare independently of or in partnership with the healthcare system” and were drawn from the American Association of Diabetes Educator's self-care behaviours (AADE-7) (47). We used the AADE-7 framework because it is an evidence-based model to promote self-management behaviours that underpin good physical health in people with diabetes and other long-term conditions (48). We did not exclude studies that reported behaviours associated with healthcare utilisation but where this was the focus of a study we mapped the behaviour against the most proximate AADE-7 behaviour. Studies that exclusively assessed adherence to psychotropic medication in people with SMI were not included as this topic has previously been reviewed (49). SMI was defined as a diagnosis of schizophrenia, affective disorders (psychotic), bipolar disorder, paranoid disorders, or psychosis (ICD codes F20–29, F30–31, F32.3, or F33.3).

In keeping with previous systematic reviews where populations with mixed diagnoses and age groups might be identified (50) we excluded studies if >70% of participants were aged over 18 years, >70% had SMI, or if the reporting of participant diagnoses was insufficient to determine eligibility. Studies with a control group of people without SMI that separately reported data from an eligible group of those with SMI were included. We included evidence from groups with or without diagnoses of long-term physical illness, with a focus on community settings. Studies of inpatient populations were excluded because they are likely to experience different determinants of self-management from individuals living in the community. Case studies, case series, conference abstracts, and dissertations were all excluded. Studies that reported on reduction or cessation of tobacco, alcohol or illicit substance use were eligible; studies that reported only on initiation or general consumption of tobacco, alcohol or illicit substances were excluded.

Because we wanted to use the findings from this systematic review to inform the development of behaviour change interventions for people with SMI and diabetes in a high-income health service context we restricted studies to those reported in English and conducted in high income countries according to 2018 OECD Country Classifications (51). There were no restrictions by date. Studies of any quantitative or mixed methods design were eligible; however experimental intervention studies were excluded because we were interested in determinants of behaviour in a naturalistic context.

### Information Sources

We searched the following databases:

- CINAHL (EBSCO) 1981- 25/07/2018
- Conference Proceedings Citation Index- Science (Clarivate Analytics Web of Science) 1990 - 25/07/2018
- Evidence Search (NICE), all available years - 25/07/2018
- HMIC Health Management Information Consortium (Ovid) 1983 - 25/07/2018
- Ovid MEDLINE(R) and Epub Ahead of Print, In-Process & Other Non-Indexed Citations and Daily 1946 to August 26, 2020
- PsycINFO (Ovid) 1806 to August Week 3 2020

We also checked relevant systematic reviews identified in the search for additional eligible primary studies.

### Search

All databases were searched on 25th July 2018. Update searches were conducted on 21st November 2019 and 27th August 2020 in the two databases that generated the most eligible studies in the original searches (MEDLINE and PsycINFO). A comprehensive search was designed using textwords, synonyms and indexing-terms. The searches were peer-reviewed by a second information specialist. An example search strategy for Ovid MEDLINE is shown in Table 2. A Medline search strategy is available as an online Supplementary Material.

**Table 2:** Ovid medline search strategy.

**Database: Ovid MEDLINE(R) <1946 to July Week 2 2018>**
1 (bipolar adj (disorder* or disease* or illness*)).tw,kf. (21737)
2 exp schizophrenia/ (97897)
3 Affective disorders, psychotic/ (2204)
4 Bipolar disorder/ (37267)
5 paranoid disorders/ (3973)
6 exp psychotic disorders/ (48180)
7 schizo*.tw,kf. (119094)
8 (mani* adj3 depress*).tw,kf. (8267)
9 (psychotic* adj3 depress*).tw,kf. (2369)
10 (severe* adj3 affective*).tw,kf. (207)
11 (severe* adj3 mental*).tw,kf. (9026)
12 (severe* adj3 depress*).tw,kf. (8801)
13 (psychos#s adj3 depress*).tw,kf. (3368)
14 (serious* adj3 affective*).tw,kf. (39)
15 “serious mood*”.tw,kf. (24)
16 (serious* adj3 mental*).tw,kf. (3957)
17 (serious* adj3 depress*).tw,kf. (616)
18 or/1-17 [Serious Mental Illness] (223362)
19 self care/ (30488)
20 self administration/ (10593)
21 Self Medication/ (4458)
22 Self Efficacy/ (17300)
23 Self-Management/ (556)
24 Self help groups/ (8641)
25 blood glucose self-monitoring/ (5606)
26 (self adj2 (efficac* or help or care* or cure* or manage* or directed or monitor* or medicat* or treat* or inject* or remed*)).tw,kf. (65658)
27 (selfefficac* or selfhelp or selfcare or selfcure* or selfmanage* or selfdirected or selfmonitor* or selfmedicat* or selftreat* or selfinject* or selfremed*).tw,kf. (185)
28 (self administ* not (self administ* adj2 (interview? or survey? or questionnaire?))).tw,kf. (15986)
29 or/19-28 [Self Management only terms] (119143)
30 life style/ or exp healthy lifestyle/ or life change events/ or sedentary lifestyle/ (81876)
31 exp Diet Therapy/ or exp Diet/ or exp Food/ or exp Feeding Behavior/ (1407344)
32 exp Exercise/ (167064)
33 smoking cessation/ or smoking reduction/ (25689)
34 Alcohol Abstinence/ (457)
35 *health promotion/ or *healthy people programs/ or *weight reduction programs/ (45563)
36 ((behavio?r or lifestyle or “life style” or habit?) adj2 (chang* or improv* or modif*)).tw,kf. (41849)
37 ((diet* or eating) adj2 (healthy or improv*)).tw,kf. (13706)
38 (physical adj1 (activit* or exercise*)).tw,kf. (89158)
39 ((Smoking or cigar* or tobacco or alcohol*) adj2 (cessation or stop* or quit* or reduc* or abstinen* or withdrawal*)).tw,kf. (42214)
40 (weight adj (loss or reduction)).tw,kf. (70109)
41 exp “treatment adherence and compliance”/ (215866)
42 ((adher* or non-adher* or compliance or non-compliance) adj2 (treatment? or medication*)).tw,kf. (21050)
43 (screening adj5 (health* or cancer*)).tw,kf. (47461)
44 or/30-43 [Healthy lifestyle] (2041439)
45 *Patient Education as Topic/ (36279)
46 exp Social Support/ (64039)
47 (social adj2 support*).tw,kf. (30139)
48 Patient care planning/ (37203)
49 or/45-48 [Patient Knowledge] (147247)
50 barrier?.ti,kf. (44604)
51 difficult*.ti,kf. (26242)
52 weakness*.ti,kf. (3988)
53 participat*.ti,kf. (31623)
54 facilitat*.ti,kf. (31063)
55 enabler*.ti,kf. (365)
56 strength*.ti,kw. (37766)
57 determinant*.ti,kf. (44930)
58 ((“Theoretical Domain?” or “Implementation Research” or Ecological or “Knowledge to Action” or “COMB B”) adj4 (Framework* or model? or system?)).tw,kf. (6077)
59 motivat*.ti,kf. (16867)
60 promot*.ti,kf. (142138)
61 goal?.ti,kf. (15048)
62 uptake.ti,kf. (62745)
63 problem?.ti,kf. (179071)
64 ((tackl* or address* or solv* or resolv* or sort*) adj1 problem*).tw,kf. (18324)
65 Problem Solving/ (23501)
66 exp Motivation/ (155294)
67 or/50-66 [Barriers or Motivators] (783038)
68 or/44,49 [Healthy lifestyle or patient knowledge] (2157107)
69 67 and 68 [Barriers to healthy lifestyle or knowledge] (125499)
70 (barriers adj4 care).ti. (1230)
71 29 or 69 or 70 [Self Management or Barriers to lifestyle change] (237043)
72 18 and 71 [SMI and SM or barriers to lifestyle change] (3836)
73 Comment/ (678448)
74 letter/ (937414)
75 editorial/ (411497)
76 note/ (1988)
77 news/ (174511)
78 newspaper article/ (18274)
79 (comment* or letter? or editorial? or note?).ti. (163738)
80 case reports/ (1881218)
81 or/73-80 (3456232)
82 Published Erratum/ or Retraction of Publication/ (5530)
83 81 not 82 [Comments/Letters] (3455452)
84 72 not 83 [SMI and SM or barriers to lifestyle change editorials etc. removed] (3531)
85 exp Animals/ not exp Humans/ (4473346)
86 (adolescent/ or child/ or infant/) not exp adults/ (1425928)
87 84 not (85 or 86) [SMI and SM or barriers to lifestyle change - editorials/children/animals removed] (3280)

### Study Selection

Unique records identified by the search were imported into Covidence (52). Two reviewers independently screened titles and abstracts and then assessed full text eligibility; conflicts were resolved in discussion or through referral to a third reviewer.

### Data Extraction

Relevant data were extracted by one reviewer into a table organised by determinants and behaviours. Using the MoA definitions (Table 3), each determinant was mapped to a MoA using descriptions reported by study authors. Some determinants were deemed to overlap with more than one MoA. We allocated evidence to the more specific MoA wherever possible. A second senior reviewer checked the extracted data and decided on determinants that had insufficient description or overlapped multiple MoAs, resulting in allocation of each data item to a single MoA.

**Table 3:** Mechanisms of action and their definition.

**Mechanism of Action**	***Definition [Reproduced from (39)]***
Knowledge	An awareness of the existence of something
Skills	An ability or proficiency acquired through practice
Social/Professional Role and Identity	A coherent set of behaviours and displayed personal qualities of an individual in a social or work setting
Beliefs about Capabilities	Beliefs about one's ability to successfully carry out a behaviour
Optimism	Confidence that things will happen for the best or that desired goals will be attained
Beliefs about Consequences	Beliefs about the consequences of a behaviour (i.e., perceptions about what will be achieved and/ or lost by undertaking a behaviour, as well as the probability that a behaviour will lead to a specific outcome)
Reinforcement	Processes by which the frequency or probability of a response is increased through a dependent relationship or contingency with a stimulus or circumstance
Intentions	A conscious decision to perform a behaviour or a resolve to act in a certain way
Goals	Mental representations of outcomes or end states that an individual wants to achieve
Memory, Attention, and Decision Processes	Ability to retain information, focus on aspects of the environment, and choose between two or more alternatives
Environmental Context and Resources	Aspects of a person's situation or environment that discourage or encourage the behaviour
Social Influences	Those interpersonal processes that can cause oneself to change one's thoughts, feelings, or behaviours
Emotion	A complex reaction pattern involving experiential, behavioural, and physiological elements
Behavioural Regulation	Behavioural, cognitive, and/or emotional skills for managing or changing behaviour
Norms	The attitudes held and behaviours exhibited by other people within a social group
Subjective Norms	One's perceptions of what most other people within a social group believe and do
Attitude toward the Behaviour	The general evaluations of the behaviour on a scale ranging from negative to positive
Motivation	Processes relating to the impetus that gives purpose or direction to behaviour and operates at a conscious or unconscious level
Self-image	One's conception and evaluation of oneself, including psychological and physical characteristics, qualities, and skills
Needs	Deficit of something required for survival, well-being, or personal fulfilment
Values	Moral, social or aesthetic principles accepted by an individual or society as a guide to what is good, desirable, or important
Feedback Processes	Processes through which current behaviour is compared against a particular standard
Social Learning/Imitation	A process by which thoughts, feelings, and motivational states observed in others are internalised and replicated without the need for conscious awareness
Behavioural Cueing	Processes by which behaviour is triggered from either the external environment, the performance of another behaviour, or from ideas appearing in consciousness
General Attitudes/Beliefs	Evaluations of an object, person, group, issue, or concept on a scale ranging from negative to positive
Perceived Susceptibility/Vulnerability	Perceptions of the likelihood that one is vulnerable to a threat

Each behaviour was then mapped to one AADE-7 category: healthy eating; being active; monitoring; taking medication; problem solving; reducing risks (e.g., smoking cessation), and healthy coping. Once a data item was mapped as described, an MoA and AADE-7 determinant-behaviour link was formed.

### Data Items

Quantitative findings describing determinants of self-management behaviours in individuals with SMI were the data of interest. Where studies included a non-SMI control group only the SMI group data were extracted.

### Quality Appraisal of Individual Studies

The methodological quality of the included studies was assessed by one reviewer using the NICE quality appraisal checklist for quantitative studies reporting correlations and associations (53), which produces separate ratings for internal and external validity. All ratings were checked by a second reviewer. We incorporated certainty of evidence in the synthesis by including cumulative ratings of internal and external validity across studies for each reported MoA and AADE-7 link. In line with GRADE ratings (54), certainty of evidence was rated as high (all positive ratings), moderate (majority positive ratings), low (balance between positive and negative ratings), and very low (all negative ratings).

### Synthesis of Results

We performed a narrative synthesis as data were too heterogenous to allow for meta-analysis of statistical tests of associations between determinants and behaviours. Studies reporting statistical tests of associations were prioritised in the synthesis. Where a study performed a multivariable analysis of determinants we opted to use the univariate associations to enhance comparability with other studies that did not include multivariate analyses. We mapped links between MoAs and AADE-7 self-management behaviours against the superordinate COM-B framework. This allowed for MoAs that derive from the TDF to be easily identifiable within the COM-B framework and offers the means to identify candidate intervention functions associated with MoAs using the behaviour change wheel (33). Links were reported as positive or negative. Where results were inconclusive we reported these as having no association.

## Results

### Study Selection

**Figure 1:**
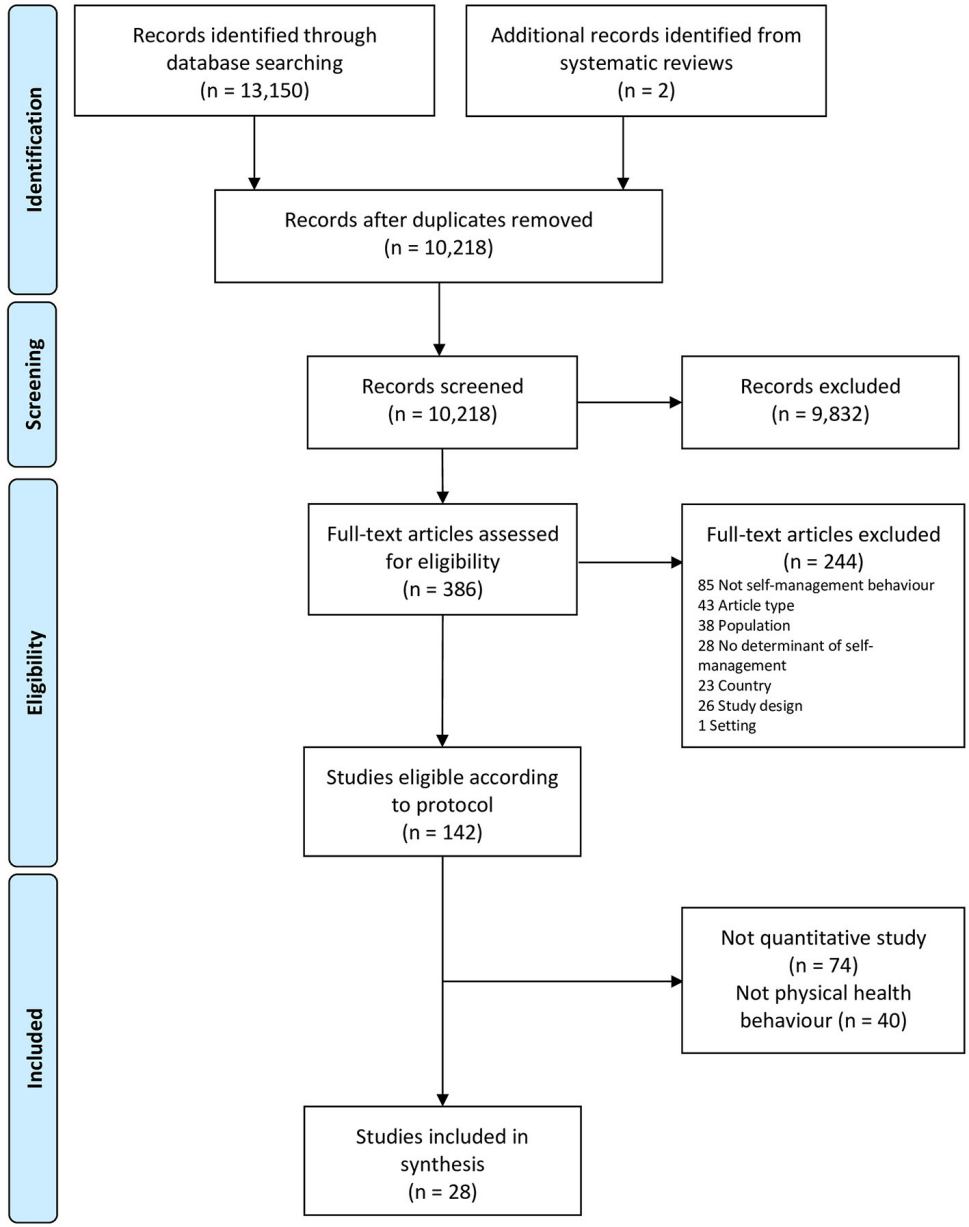
PRISMA flowchart.

A flowchart of study selection in the context of the overarching review is shown in Figure 1. Of the 10,218 unique studies identified from searches, 386 were assessed as potentially eligible based on titles and abstracts and 28 studies were included.

### Study Characteristics

Characteristics of the included studies are shown in Table 4. Twenty-four studies were of people with SMI and four studies were of people with SMI and diabetes (63, 69, 71, 77). We did not identify any study that met eligibility criteria that included populations of people with SMI and other long-term physical conditions. There were no studies of the perspective of clinicians or carers about determinants for individuals with SMI. Twenty-six studies used a cross-sectional design and two used a prospective cohort design. Nine studies were conducted in the USA, five in the UK, four in Canada, two each in Australia and Belgium, one each in Israel, Ireland, Italy, Japan and the Netherlands, and one study in both the Netherlands and Belgium.

**Table 4:** Characteristics of included studies.

**Study ID Design, sample size**	**Country setting**	**SMI diagnoses of study sample**	**Mean age % female**	**Ethnicity**
Arbour-Nicitopoulos et al. (55)Prospective cohort, *N* = 101	Canada Community setting	Schizophrenia 67.3% Schizoaffective 31.7% Psychosis not otherwise specified 1.0%	41.5 years 40.6%	White 57.4% African 18.8% South Asian 5.9% Asian 6.9% Other 10.9%
Ashton et al. (56) Cross-sectional, *N* = 1,043	Australia Smoking cessation programme within adult MH services	Schizophrenia 37.1% Depression and/or anxiety 20.5% Bipolar disorder 18.3% Schizoaffective disorder 16.7% Other* 7.4% (percentages are of *n* = 868 with a recorded diagnosis)	41.9 years (men) 45.1 years (women) 49.6%	NR
Berti et al. (57) Cross-sectional, N = 193	Italy Community MH service	Schizophrenia or related psychosis 78% Affective psychosis 22%	48 years 47%	NR
Bezyak et al. (58) Cross-sectional, *N* = 92	USA Outpatient treatment program	Schizophrenia 55.8% Schizoaffective disorder 33.7% Bipolar disorder 6.3% Other mood disorders 3.2% Other psychotic disorders 1.1%	Range 18–70 years 25.3%	NR
Campion et al. (59) Cross-sectional, *N* = 43	UK Rehabilitation centre and outpatient depot clinic	“The majority had a formal diagnosis of schizophrenia”	52.6 years 39%	NR
Dickerson et al. (60) Cross-sectional, *N* = 78	USA Outpatient MH services	Schizophrenia or schizoaffective disorder 64% Bipolar disorder 22% Major depression 13% Other 1%	50.0 years 40%	Caucasian 72% African American 27% Other 1%
Faulkner et al. (61) Cross-sectional, *N* = 109	Canada Smoking cessation clinic within an MH and Addiction facility	Currently receiving treatment for mental health disorders (e.g., schizophrenia or depression) 92.7% Did not respond 6.3%	46.5 years 46.8%	White 91.0% Black/Asian 3.6% Aboriginal 1.8% Hispanic 0.9% Not identified 2.7%
Filia et al. (62) Cross-sectional, *N* = 43	Australia Community MH settings	Schizophrenia 53.5% Schizoaffective disorder 25.6% Bipolar affective disorder 13.9% Other non-organic psychotic syndrome 7.0%	36.3 years 41.9%	Australian born 90.7%
Gorczynski et al. (63) Cross-sectional, *N* = 63	Canada Outpatient psychiatric facility	Schizophrenia 52.4% Schizoaffective disorder 23.8% Bipolar 1 disorder 17.5% Major depressive disorder with psychosis 3.2% Other forms of psychosis 3.1%	50.2 years 38.1%	White 58.7% Black 19.0% Asian 12.7%
Kelly et al. (64) Cross-sectional, *N* = 100*	USA Psychiatric research centre outpatient and inpatient programs	Individuals with a diagnosis of schizophrenia or schizoaffective disorder recruited but characteristics of sample not reported	43.3 years 29%	Caucasian 61% African American 37% Other 2%
Klingaman et al. (65) Cross-sectional, *N* = 5,388	USA Veterans Affairs weight management program	Schizophrenia 100%	Median 55 years 14.1%	**Race** White 49.2% Black 32.2% Other 5.5% Unknown 13.1% **Ethnicity** Hispanic 11.3%
Kreyenbuhl et al. (66) Cross-sectional, *N* = 44*	USA Public and private outpatient MH clinics in urban and suburban communities	Schizophrenia-spectrum disorder 70% Major mood disorder 30%	51.1 years 55%	Non-Caucasian 41%
Matthews et al. (67) Cross-sectional, *N* = 105	Ireland Rehabilitation and recovery mental facilities	Schizophrenia 44% Affective disorder 39% Majority of sample taking antipsychotic medication (70%)	52 years 29%	NR
Mishu et al. (68) Cross-sectional, *N* = 3,287	UK Large SMI research cohort recruited from primary and secondary care	Individuals with diagnosis of schizophrenia or other psychotic disorders, bipolar disorder or depression with psychotic features eligible for study but characteristics of sample not reported	47.7 years 40.1%	White British 85.7% Other 14.3%
Mulligan et al. (69) Cross-sectional, *N* = 77	UK National Health Service, charities and service user networks	Schizophrenia 36.4% Schizoaffective disorder 16.9% Depression with psychotic features 22.1% Bipolar disorder 41.6% (may have more than one diagnosis)	52.3 years 46.8%	**Ethnicity** White, British 61.0% White, other 9.1% South Asian 10.4% Black African Caribbean 7.8% Other 10.4% Missing 1.3% Missing 24.7%
Muralidharan et al. (70) Cross-sectional, *N* = 17,826	USA Veterans Affairs weight management program	Schizophrenia or bipolar disorder (proportions not reported)	54 and younger 49.3% 55 and older 50.7% 21.3%	**Race** White 59.9% Black 23.7% Other 4.9% Unknown/missing 11.6% **Ethnicity** Hispanic 9.2%
Ogawa et al. (71) Cross-sectional, *N* = 38	Japan Outpatient psychiatric care	Schizophrenia 100%	53.9 years 39.5%	NR
Peckham et al. (72) Cross-sectional, *N* = 97	UK Primary and secondary care	Schizophrenia or other psychotic illness 59% Bipolar disorder 31% Schizoaffective disorder 10%	Median 47.2 40.2%	NR
Prochaska et al. (73) Cross-sectional, *N* = 685	USA Online mood disorder peer-support network	Bipolar disorder 100%	26–50 years 67% Not reported 33% 67%	Non-Hispanic Caucasian 89% Not reported 11%
Romain and Abdel-Baki (74) Cross-sectional, *N* = 43	Canada Physical activity programme (baseline data)	Schizophrenia 30.2% Bipolar disorders 23.3% Psychosis other 20.9% Schizo-affective disorders 14% Major depressive disorders 9.3% Severe anxiety disorders 2.3%	29.0 years 32.3%	NR
Roosenschoon et al. (75) Cross-sectional, *N* = 187	Netherlands Outpatient MH services	Psychotic disorders 57% Mood disorder 33% Personality disorder 35% (Total >100% because participants had multimorbidity)	44.3 years 47%	NR
Shor and Shalev (76) Cross-sectional, *N* = 86	Israel Community MH facilities	Schizophrenia 70% Bipolar disorder 16% Depression 14%	39.4 years 52%	NR
Spivak et al. (77) Cross-sectional, *N* = 271	USA Inner-city outpatient MH centres	Primary diagnosis Schizophrenia 33% Mood disorder with psychotic features 60% Psychotic disorder not otherwise specified 7%	42 years 53%	Black 54% White 34% Other 12%
Twyford and Lusher (78) Cross-sectional, *N* = 105*	UK Community MH services	Schizophrenia 100%	38.3 years 21%	NR
Vancampfort et al. (79) Cross-sectional, *N* = 69*	Belgium Psychiatric centres	Schizophrenia 100%	40.6 years 34.1%	NR
Vancampfort et al. (80) Cross-sectional, *N* = 29*	Belgium Psychiatric centres	First-episode psychosis 100%	Men mean 25.0 years Women mean 23.7 years 35.7%	NR
Vermeulen et al. (81) Prospective cohort, *N* = 1,094	Netherlands and Belgium University medical centres and associated mental health-care institutions	Schizophrenia 66% Others not reported	NR	NR
Zechner and Gill (82) Cross-sectional, *N* = 120	USA Outpatient MH services	Bipolar disorder 35.0% Schizophrenia 29.2% Schizoaffective disorder 8.3% Depression 35.8% (Total >100% because participants had multimorbidity)	47.5 years 40%	Black 50.8% White 37.5% Hispanic 5.0% American Indian 1.7% Asian 1.7% Other 3.3%

Table 5 shows the links between outcomes and AADE-7 self-management behaviours and between measured determinants and MoAs across all included studies. Six studies reported determinants of multiple self-management behaviours, two of which focused on a range of diabetes self-management activities (69, 71); the other four reported behaviours including physical activity, healthy eating, reducing risks (smoking cessation and alcohol consumption) (57, 59, 61, 65). Of the studies that focused on a single behaviour, eleven reported determinants of being active (55, 58, 63, 67, 68, 70, 74, 76, 78–80, 82), seven were about reducing risks [smoking cessation (56, 60, 62, 64, 72, 73, 81), seeking professional help (77), alcohol or drug use (75)], and one was about taking medications (66). Studies reported evidence aligning with a mean of five different MoAs (range 1–14) and there was evidence identified for 21 of 26 MoAs. The links between reported health outcomes and AADE-7 self-management behaviours and the links between reported determinants and MoAs are shown in Table 3.

**Table 5:** Links between outcomes and AADE7 self-management behaviours and between measured determinants and MoAs.

**Study IDDesign, sample size**	**Outcome measure**	**AADE7 Behaviour (s)**	**Determinant (s) and measures**	**Mechanism of Action**
Arbour-Nicitopoulos et al. (55)	Accelerometer	Physical activity	Intentions Action planning Coping planning Maintenance self-efficacy Health Action Process Approach inventory for adults with schizophrenia Social support Multidimensional Scale of Perceived Social Support	Intentions Behavioural cueing Behavioural regulation Beliefs about Capabilities Environmental context and resources
Ashton et al. (56)	Pre-programme questionnaire for the Tobacco Free programme	Reducing risks – smoking cessation	Fitness; Impact on others Financial reasons Wanting freedom from addiction Health concerns Stigma Questionnaire (origin not reported)	Beliefs about consequences Environmental context and resources Goals Perceived susceptibility/vulnerability Subjective norms
Berti et al. (57)	PASSI Questionnaire developed for the PASSI project	Physical activity Reducing risks—smoking cessation; alcohol consumption	Employment status Client Sociodemographic and Service Receipt Inventory (CSSRI-EU)	Environmental context and resources
Bezyak et al. (58)	The Physical Activity Scale for Individuals with Physical Disabilities (PASIPD), modified to refer to mental illness	Physical activity	Self-efficacy Multidimensional Self-Efficacy Questionnaire (MSEQ) Perceived pros and cons Decisional Balance for Exercise Adoption Relationships with program staff An exploratory question asked whether relationships with treatment program staff encouraged them to be physically active	Beliefs about capabilities Beliefs about capabilities Social influences
Campion et al. (59)	Reported Health Behaviours Checklist. Interviews (yes/no questions) based on stages of change model	Physical activity Reducing risks—smoking cessation	Time; opportunity; expense Enjoyment; stress reduction Doctor's advice Social activity Prevent health problems Injury/weakness Media influence Questions based on dimensions of the Health Belief Model Interest in the behaviour Time of day	Environmental context and resources Reinforcement Social influences Needs Perceived susceptibility/vulnerability Beliefs about capabilities Subjective norms Motivation Behavioural cueing
Dickerson et al. (60)	Structured interviews relating to smoking cessation	Reducing risks—smoking cessation	Health concerns Cost of cigarettes; experience of smoking restrictions in hospital; smoking restrictions at home or work Suggestion or advice from a doctor; suggestion or advice from others Example of family members who had died from a smoking-related illness; example of friends who quit Structured interview developed by authors	Perceived susceptibility/vulnerability Environmental context and resources Social influences Social learning/imitation
Faulkner et al. (61)	Survey assessing perceived interest in physical activity Number of attempts to quit smoking	Physical activity Reducing risks - smoking cessation	Various perceived advantages of (e.g., it would improve my muscle tone) and barriers to (e.g., it would leave me feeling tired) becoming more physically active Improve how I feel about myself Improve my health or reduce my risk of disease; I might injure myself I would have to do it by myself; it would take time away from other things; it would cost too much I would worry about what other people think of me; I would be worried that I would not be very good at it I don't know how to do it It would be difficult to find out what to do and where to do it Decisional balance measure devised for the study Interest in physical activity (as a determinant of smoking cessation)	Beliefs about consequences Self-image Perceived susceptibility/vulnerability Environmental context and resources Emotion Knowledge Beliefs about capabilities Motivation
Filia et al. (62)	Opiate treatment index (OTI) Fagerstrom Test for Nicotine dependence Breath levels of carbon monoxide	Reducing risks—smoking cessation	Health concerns Self-control (to prove I can; to feel in control; can do other things) Reinforcement (I won't smell; to save money; won't burn holes in clothes) Social influence Reasons for quitting questionnaire Stress reduction (relaxation; to take a break; reduce stress); addiction (habit; craving); arousal (peps me up; weight control; enjoyment; to help concentration); mental illness	Perceived susceptibility/vulnerability Self-image Beliefs about consequences Social influences
			Partner smoking Reasons for smoking questionnaire	Reinforcement Behavioural cueing
Gorczynski et al. (63)	International Physical Activity Questionnaire (IPAQ)	Physical activity	Self-efficacy Perceived barriers Perceived benefits Patient-Centered Assessment and Counseling for Exercise (PACE) questionnaire Negative symptoms of psychosis (alogia; affective blunting; avolition-apathy; anhedonia-asociality; attentional impairment) Scale for the Assessment of Negative Symptoms (SANS)	Beliefs about capabilities Attitude toward the behaviour Beliefs about consequences Motivation
Kelly et al. (64)	Fagerström Test for Nicotine Dependence	Reducing risks—smoking cessation	Health concerns Self-control (to prove I can; to feel in control; can do other things) Reinforcement (I won't smell; to save money; won't burn holes in clothes)	Perceived susceptibility/vulnerability Self-image
			Social pressure Reasons for quitting questionnaire	Beliefs about consequences Social influences
Klingaman et al. (65)	MOVE!23, a multidimensional assessment of factors related to weight management.	Physical activity Diet	Eating from restaurants/convenience stores/vending machines; too much high calorie food at home/work; too little time to prepare and eat healthy food; too little money to buy healthy food; job/work schedule; no place to walk/be active; no transportation; lack of support/encouragement	
			Feeling hungry much of the time; too tired Used to eating a certain way Stress/depression Being with others who overeat Safety concerns Do not like the behaviour Daily routines “MOVE!23,” a multidimensional assessment of factors related to weight management	Environmental context and resources Motivation Behavioural regulation Emotion Subjective norms Perceived susceptibility/vulnerability Attitude toward the behaviour Behavioural cueing
Kreyenbuhl et al. (66)	Brief Medication Questionnaire	Medication adherence	Motivation barrier (how well does your diabetes medication work for you?) Recall barrier Access barrier Brief Medication Questionnaire Beliefs about the necessity of diabetes medications for controlling the illness; concerns about the adverse consequences of taking the medications	Beliefs about consequences Memory, attention, and decision processes Environmental context and resources
			Beliefs about Medication Questionnaire: Specific Version (BMQ-Specific) HbA1c	Beliefs about consequences Behavioural Regulation
Matthews et al. (67)	3Q physical activity assessment	Physical activity	Environmental resources Motivation and goals Beliefs about capabilities Knowledge Skills Emotion Social influences Beliefs about the consequences Action planning Coping planning Goal conflict The Determinants of Physical Activity Questionnaire (DPAQ)	Environmental context and resources Motivation, Goals Beliefs about capabilities Knowledge Skills Emotion Social influences Beliefs about consequences Behavioural cueing Behavioural regulation Goals
Mishu et al. (68) Cross-sectional	Self-reported participation in regular physical activity	Physical activity	Employment status Self-rated health Health problem limiting activity Importance of maintaining healthy lifestyle Would like to take more exercise Closing the Gap: The Lifestyle Health and Well-being (HWB) survey	Environmental context and resources Self-image Beliefs about capabilities Values Attitude toward the behaviour
Mulligan et al. (69) Cross-sectional	Summary of Diabetes Self-Care Activities (SDSCA)	Control—self-monitoring of blood glucose Medication adherence General diet Specific diet (eating fruit and vegetables and high fat foods) Physical activity Reducing risks—checking feet; smoking	Psychological distress CORE-10 Knowledge Skills Social/professional role and identity Beliefs about capabilities; most difficult self-management behaviour Optimism Beliefs about consequences Reinforcement Intentions Goals Memory, attention and decision processes Environmental context and resources; employment status Social influences Emotion Behavioural regulation Questionnaire based on the Theoretical Domains Framework (TDF)	Emotion Knowledge Skills Social/Professional role and identity Beliefs about capabilities Optimism Beliefs about consequences Reinforcement Intention Goals Memory, attention an decision processes Environmental context and resources Social influences Emotion Behavioural regulation
Muralidharan et al. (70) Cross-sectional	“MOVE!23,” a multidimensional assessment of factors related to weight management	Physical activity	Physical health barriers Too little money; lack of support/encouragement; too little time; job/work schedule; no transportation; no place to walk/be active Too tired Stress/depression Safety concerns Do not like the behaviour Daily routines “MOVE!23,” a multidimensional assessment of factors related to weight management	Beliefs about capabilities Environmental context and resources Motivation Emotion Perceived susceptibility/vulnerability Attitude toward the behaviour Behavioural cueing
Ogawa et al. (71)	Summary of Diabetes Self-Care Activities Questionnaire—Japanese version (SDSCA-J)	Physical activity Medication adherence Control - blood sugar testing; foot care	Symptom severity Brief Psychiatric Rating Scale (BPRS)	Emotion
Cross-sectional			Psychosocial functioning Global Assessment of Functioning Scale (GAF)	Skills
	Food Frequency Questionnaire Based on Food Groups (FFQg)	Diet	HbA1c Most recent test in medical records	Behavioural regulation
Peckham et al. (72) Cross-sectional	Standardised interview about reasons for smoking and wanting to quit	Reducing risks–smoking cessation	Helps to cope with stress; helps to relax; bad for my health; makes me less fit; bad for the health of people near me; bad example for children; unpleasant for people near me; makes my clothes and breath smell; I enjoy it; breaks up working time	Beliefs about consequences
			Something to do when bored Stops withdrawal symptoms Something to do with friends Stops me putting on weight I don't like feeling dependent on cigarettes Expense People around me disapprove Standardised interview about reasons for smoking and wanting to quit	Behavioural cueing Reinforcement Social influences Goals Self-image Environmental context and resources Subjective norms
Prochaska et al. (73) Cross-sectional	Survey developed for the study about tobacco use, attempts to quit and tobacco-related attitudes and intentions	Reducing risks—smoking cessation	Gets in the way of living the life that I want Stress; craving Tobacco use by family and friends; support from family and friends; encouragement from healthcare providers Belief I can quit Pleasure of behaviour; concerns it would make my mental illness worse To treat my mental illness; being in good mental health Survey developed for the study about tobacco use, attempts to quit and tobacco-related attitudes and intentions	Goals Emotion Environmental context and resources Beliefs about capabilities Beliefs about consequences Motivation
Romain and Abdel-Baki (74)Cross-sectional	Global Physical Activity Questionnaire	Physical activity	Self-efficacy French translation of a scale developed by Bandura (83) Perceived advantages and inconveniences of the behaviour Decisional Balance Scale for Exercise	Beliefs about capabilities Beliefs about consequences
Roosenschoon et al. (75)Cross-sectional	Single item from Addiction Severity Index (ASI)	Reducing risks—problems with alcohol/drug use	Social support Multidimensional Scale of Perceived Social Support (MSPSS)	Environmental context and resources
Shor and Shalev (76)Cross-sectional	Scales designed to measure participants' perception of the barriers to and benefits of involvement in physical activities	Physical activity	Knowledge Accessibility Thoughts that the behaviour will not improve the person's condition; fear that the behaviour will have a negative effect on one's health; improvement of mental health and health Improvement of feelings about body Side effects of psychiatric medications; weight and associated co-morbid health problems Mental health Scales designed to measure participants' perception of the barriers to and benefits of involvement in physical activities	Knowledge Environmental context and resources Beliefs about consequences Self-image Beliefs about capabilities Emotion
Spivak et al. (77)Cross-sectional	Delays in seeking medical care over the past 12-month period were assessed by using seven items, five of which were derived from the National Health Interview Survey	Reducing risks—seeking professional help	Couldn't get through on the telephone; couldn't get an appointment soon enough; once you get there you have to wait too long to see a doctor; the (clinic/doctor's) office wasn't open when I could get there; didn't have transportation; did not have health insurance or could not afford to receive care Concerns about being treated differently because of mental illness Questions partly derived from the National Health Interview Survey	Environmental context and resources Subjective norms
Twyford and Lusher (78)Cross-sectional	A questionnaire adapted from Godin and Shephard's (84) Leisure-Time Activity Questionnaire (GLTEQ)	Physical activity	Behavioural beliefs Attitudes Subjective norms Perceived behavioural control; Self-efficacy Intention A questionnaire adapted from Ajzen's (85) guide to constructing a Theory of Planned Behaviour questionnaire Living situation; employment status; health professional support	Beliefs about consequences Attitude toward the behaviour Subjective norms Beliefs about capabilities Intentions Environmental context and resources
Vancampfort et al. (79)Cross-sectional	Behavioural Regulation in Exercise Questionnaire 2 (BREQ-2)	Physical activity	Amotivation External regulation Introjected regulation Autonomous regulation Behavioural Regulation in Exercise Questionnaire 2 (BREQ-2)	Motivation Social influences Values Reinforcement
Vancampfort et al. (80)Cross-sectional	Patient-Centred Assessment and Counseling for Exercise	Physical activity	Amotivation External regulation Introjected regulation Autonomous regulation Behavioural Regulation in Exercise Questionnaire 2 (BREQ-2)	Motivation Social influences Values Reinforcement
Vermeulen et al. (81)Prospective cohort	Composite International Diagnostic Interview (CIDI)	Reducing risks—smoking initiation and cessation	Symptom frequency Community Assessment of Psychotic Experience (CAPE) Symptom severity Emotional distress Positive And Negative Syndrome Scale (PANSS) Quality of life WHO Quality of Life (WHOQOL) schedule	Motivation Motivation Emotion Self-image
Zechner and Gill (82)Cross-sectional	International Physical Activity Questionnaire—Short Form (IPAQ)	Physical activity	Social support Social Support for Exercise Scale Self-efficacy Self-efficacy for Exercise Scale Outcome expectations Outcome Expectations for Exercise Scale Goal-setting, self-monitoring, problem solving Exercise Goal-Setting Scale Psychological distress due to psychiatric symptoms Brief Symptom Inventory	Environmental context and resources Beliefs about capabilities Beliefs about consequences Behavioural regulation Emotion

### Quality Appraisal of Individual Studies

Quality appraisal ratings are shown in Table 6. Five studies were rated as having high internal validity (55, 63, 71, 77, 81), but no studies were rated as having both high internal and external validity. Six studies were rated as having both low internal and low external validity (56, 59, 61, 62, 65, 76).

**Table 6:** Quality appraisal ratings for individual studies.

	**Population**			**Method of selection of exposure (or comparison) group**					**Outcomes**					**Analyses**				**Summary**	
**References**	**1.1 Is the source population or source area well-described?**	**1.2 Is the eligible population or area representative of the source population or area?**	**1.3 Do the selected participants or areas represent the eligible population or area?**	**2.1 Selection of exposure (and comparison) group. How was selection bias minimised?**	**2.2 Was the selection of explanatory variables based on a sound theoretical basis?**	**2.3 Was the contamination acceptably low?**	**2.4 How well were likely confounding factors identified and controlled?**	**2.5 Is the setting applicableto the UK?**	**3.1 Were the outcome measures and procedures reliable?**	**3.2 Were the outcome measurements complete?**	**3.3 Were all the importantoutcomes assessed?**	**3.4 Was there a similar follow-up time in exposure and comparison groups?**	**3.5 Was follow-up meaningful?**	**4.1 Was the study sufficiently powered to detect an intervention effect (if one exists)?**	**4.2 Were multiple explanatory variables considered in the analyses?**	**4.3 Were the analytical methods appropriate?**	**4.4 Was the precision of association given or calculable? Is association meaningful?**	**5.1 Are the study results internally valid (i.e., unbiased)?**	**5.2 Are the findings generalisable to the source population (i.e., externally valid)?**
Arbour-Nicitopoulos et al. (55)	+	NR	+	NA	++	NA	+	+	++	-	NA	+	++	NA	++	++	++	++	+
Ashton et al. (56)	++	–	–	NA	++	NA	–	+	–	–	NA	NA	NA	NA	–	–	–	–	–
Berti et al. (57)	++	++	+	NA	+	NA	–	+	–	NR	NA	NA	NA	NA	+	+	–	+	+
Bezyak et al. (58)	+	+	+	NA	++	NA	NA	–	–	+	NA	NA	NA	NA	–	–	–	–	+
Campion et al. (59)	+	–	–	NA	++	NA	NR	+	–	NR	NA	NA	NA	NA	+	NR	–	–	–
Dickerson et al. (60)	+	+	–	NA	–	NA	–	+	–	+	NA	NA	NA	NA	–	–	–	–	+
Faulkner et al. (61)	–	–	–	NA	–	NA	–	+	–	+	NA	NA	NA	NA	–	–	–	–	–
Filia et al. (62)	+	+	–	NA	+	NA	–	+	–	++	NA	NA	NA	NA	–	–	–	–	–
Gorczynski et al. (63)	–	NR	NR	NA	++	NA	+	+	+	++	NA	NA	NA	NA	++	+	++	++	–
Kelly et al. (64)	+	+	+	NA	+	NA	–	+	–	NR	NA	NA	NA	NA	–	–	–	–	+
Klingaman et al. (65)	+	–	+	NA	NR	NA	NR	–	–	–	NA	NA	NA	NA	–	–	–	–	–
Kreyenbuhl et al. (66)	+	–	+	NA	++	NA	NA	–	–	++	NA	NA	NA	NA	++	+	++	+	+
Matthews et al., (67)	+	+	–	NA	++	NA	–	+	+	++	NA	NA	NA	NA	–	+	–	+	+
Mishu et al. (68)	+	+	+	NA	NR	NA	–	++	–	–	NA	NA	NA	NA	++	+	++	+	+
Mulligan et al. (69)	+	++	+	NA	++	NA	–	++	–	++	NA	NA	NA	NA	++	+	++	+	+
Muralidharan et al. (70)	+	+	+	NA	+	NA	NA	–	–	++	NA	NA	NA	NA	+	–	–	–	+
Ogawa et al. (71)	+	+	+	NA	+	NA	++	+	+	+	NA	NA	NA	NA	++	++	++	++	+
Peckham et al. (72)	+	+	+	NA	NR	NA	–	++	–	–	NA	NA	NA	NA	+	–	–	–	+
Prochaska et al. (73)	+	+	+	NA	–	NA	–	+	–	+	NA	NA	NA	NA	–	–	–	–	+
Romain and Abdel–Baki (74)	–	NR	+	NA	++	NA	NA	+	NR	++	NA	NA	NA	NA	++	–	+	+	–
Roosenschoon et al. (75)	+	+	+	NA	++	NA	–	+	–	++	NA	NA	NA	NA	+	+	+	+	+
Shor and Shalev (76)	+	+	–	NA	+	NA	–	+	–	+	NA	NA	NA	NA	–	–	+	–	–
Spivak et al. (77)	+	+	+	NA	NR	NA	++	–	–	+	NA	NA	NA	NA	++	++	++	++	+
Twyford and Lusher (78)	+	–	–	NA	++	NA	NA	++	–	NR	NA	NA	NA	NA	++	–	+	+	–
Vancampfort et al. (79)	+	+	NR	NA	++	NA	NA	+	–	++	NA	NA	NA	NA	–	–	–	–	+
Vancampfort et al. (80)	+	+	+	NA	++	NA	–	+	+	++	NA	NA	NA	NA	+	++	++	+	+
Vermeulen et al. (81)	–	+	+	NA	++	NA	++	+	+	+	NA	+	+	NA	++	+	++	++	+
Zechner and Gill (82)	–	–	NR	NA	++	NA	+	–	+	–	NA	NA	NA	NA	+	+	+	+	+

### Synthesis of Findings About Links Between MoAs and AADE-7 Self-Management Behaviours

Twenty-one MoAs were identified as determinants of self-management behaviours for people with SMI and people with SMI and diabetes. Table 7 reports evidence of positive (green), negative (red), and no significant associations (amber) between MoAs and AADE-7 self-management behaviours. MoAs are grouped under the super-ordinate categories used in the COM-B framework.

**Table 7:** Associations between mechanisms–of–action and AADE-7 self-management behaviours.

**COM-B: Mechanism of Action**	**Positive Association**	**Certainty of evidence**	**Negative Association**	**Certainty of evidence**	**Inconclusive**	**Certainty of evidence**
**Capability**						
Knowledge	Healthy eating (69)	Moderate			Being active (67)	Moderate
Memory, attention, and decision processes			Healthy eating (69)	Moderate		
Behavioural regulation	Monitoring, problem solving, being active (82)	Moderate	Healthy eating, monitoring (69)	Moderate	Being active (55, 67); taking medications (66)	Moderate
Skills					Being active (67, 71); Monitoring (69, 71); taking medication, healthy eating, reducing risks (71)	Moderate
**Opportunity**						
Social influences	Healthy eating, being active (69)	Moderate			Being active (67); Monitoring, taking medication, reducing risk (69)	Moderate
Environmental context and resources	Being active (69, 82); healthy eating, taking medication	Moderate	Being active (55)	Moderate	Being active (67, 78); Monitoring, reducing risks (69)	Moderate
	(69); reducing risk (75)					
Behavioural cueing					Being active (55, 67)	Moderate
**Motivation**						
Reinforcement	Healthy eating, being active (69)	Moderate			Monitoring, taking medication, reducing risks (69)	Moderate
Emotion	Reducing risks (69, 71, 81); being active (69, 82); healthy eating, monitoring (69)	Moderate			Being active (67)	Moderate
Beliefs about capabilities	Being active (55, 58, 61, 63, 68, 69, 74, 78, 82); healthy eating (69)	Moderate			Being active (67)	Moderate
Beliefs about consequences	Being active (58, 61, 67, 74, 78, 82); healthy eating (69)	Low to Moderate			Monitoring, taking medication, reducing risks (69)	Moderate
Motivation	Being active (67); Reducing risks (61)	Low to Moderate	Being active (63)	Moderate	Reducing risks (81)	Moderate
Intentions	Being active (69, 78); healthy eating (69)	Moderate			Monitoring; taking medication; reducing risks (69)	Moderate
Goals	Healthy eating (69); being active (67, 69)	Moderate	Taking medication, monitoring, reducing risks (69)	Moderate		
Subjective norms	Being active (78)	Low				
Attitude toward the behaviour			Being active (63)	Moderate	Being active (68)	Moderate
Self-image	Being active (61, 68)	Low			Reducing risks (81)	Moderate
Values	Being active (68)	Moderate				
Perceived susceptibility/vulnerability	Being active, reducing risks (61)	Very low				
Optimism	Healthy eating (69)	Moderate			Being active, monitoring; taking medication; reducing risks (69)	Moderate
Social/Professional Role and Identity					Monitoring, healthy eating, taking medication; being active, reducing risks (69)	Moderate

*Green highlighted text indicates a positive association between mechanism-of-action and self-management behaviour; red highlighted text indicates a negative association between mechanism-of-action and self-management behaviour; and amber highlighted text indicates that the evidence was inconclusive to determine an association between a mechanism-of-action and a self-management behaviour*.

#### Capability

Nineteen tests of association between MoAs and self-management behaviours were identified in six studies that could be grouped under the Capability domain. There was only limited evidence from one cross-sectional study about barriers to effective diabetes management in people with SMI, which reported that knowledge was positively associated with the frequency of following a healthy eating plan (69). This same study showed that memory, attention and decision processes and behavioural regulation were negatively associated with healthy eating. A non-significant association in either direction was observed for skills in relation to healthy eating.

There was mixed evidence that behavioural regulation was associated with monitoring, with one result showing a positive association with this behaviour (82), and another reporting a negative association (69). Evidence that skills are associated with monitoring was equivocal, with no association between this MoA and behaviour reported in two cross-sectional studies (69, 71). Additionally, memory, attention, and decision processes were not reported as being significantly associated with monitoring.

Behavioural regulation was positively associated with being active in a cross-sectional study of predictors of physical activity in people with a wide range of SMI (82); however there was no evidence of association between this MoA and behaviour in a longitudinal study of physical activity intentions in people with schizophrenia (55). There was descriptive evidence that lack of knowledge about how to do physical activities was the third highest ranked of eight barriers to being active (76), but another cross-sectional study found no association between knowledge and physical activity (67). Memory, attention, and decision processes were not significantly associated with being active (69).

Cross-sectional data from a study about glycaemic control and diabetes self-care in people with schizophrenia did not show either a positive or negative relationship between skills and behaviours associated with reducing risks (71). Memory, attention, and decision processes were also not significantly associated with reducing risks (69). Neither memory, attention, and decision processes (69), behavioural regulation (66), or skills (71) were significantly associated with taking diabetes medication (71). Furthermore, memory, attention, and decision processes were cited as a barrier among 75% of participants in a comparative cross-sectional study of taking diabetes medications in people with and without SMI (66). Only one significant association was observed for problem solving, with one study showing a positive association between behavioural regulation and this behaviour (82).

#### Summary of Findings for Capability

The certainty of evidence for associations between MoAs and AADE-7 health behaviours within the Capability domain was rated as moderate across all studies. Only two studies reported positive associations: one for healthy eating (knowledge) and one for monitoring, problem solving, and being active (behavioural regulation). One study reported negative associations for healthy eating with memory, attention and decisional processes and behavioural regulation and also with monitoring for behavioural regulation. The majority of associations in this domain were inconclusive for five of the seven health behaviours.

#### Opportunity

Eighteen tests of association between MoAs and self-management behaviours were identified in studies that could be grouped under the Opportunity domain. Cross-sectional data from one study showed that social influences and environmental context and resources were positively associated with healthy eating (69). Environmental context and resources, defined as aspects of the situation and surroundings that influence engagement in health behaviours, were also implicated in predicting physical activity. Evidence from two studies showed that social support and support from health professionals was positively associated with being active in people with SMI (82) and also in people with SMI and diabetes (69). Data from a Canadian prospective cohort study (55) showed that support from family, friends, and significant others was not associated with physical activity and a UK cross-sectional study (78) showed health professional support explained variance in exercise intention but not behaviour in people with schizophrenia. There was evidence from multiple studies that physical activity was more frequent in the employed than the unemployed (57, 68, 69, 78). Additionally behavioural cuing was not significantly associated with being active (55, 67). Tests of associations between monitoring and MoAs were observed in only one study. Mulligan et al. showed that social influences and environmental context and resources were not significantly associated with monitoring in a population with SMI (69). Reducing risks associated with alcohol and drug use was positively associated with environmental context and resources in one cross-sectional study in people with a range of psychotic and mood disorders and personality disorder (75). There were more equivocal findings in one other study which found no evidence for a significant association between either environmental context and resources or social influences and reducing risks (69). There was scant evidence of associations between MoAs aligned with Opportunity and taking medications. Findings from one cross-sectional study showed that access to health services was positively associated with taking diabetes medication, suggesting that environmental context and resources are important drivers of this behaviour (69).

#### Summary of Findings for Opportunity

The certainty of evidence for associations between MoAs and AADE-7 health behaviours within the Opportunity domain was rated as moderate across all studies. There was more inconclusive evidence for the importance of social influences being associated with behaviours, with only one study showing a positive association for this MoA with healthy eating and being active. The role of environmental context and resources appears to be important with four studies reporting positive associations for four behaviours (being active; healthy eating; taking medication; and reducing risk). Two studies reported negative associations with being active and environmental context and resources. There was little conclusive evidence about the role of behavioural cueing in prompting behaviours.

#### Motivation

Sixty-three tests of association between MoAs and self-management behaviours were identified in twelve studies that could be grouped under the Motivation domain. The most consistent evidence was observed between MoAs and healthy eating, with positive associations observed in one cross-sectional study for reinforcement, emotion, beliefs about capabilities, beliefs about consequences, intentions, goals, and optimism (69). This study also reported non-significant associations between healthy eating and social/professional role identity.

Evidence about the links between MoAs and monitoring was also drawn from the same cross-sectional study, but findings were equivocal. A positive significant association was reported for the link between emotion and monitoring, but no significant associations were observed for reinforcement, beliefs about consequences, intentions, goals, optimism, and social/professional role identity with this behaviour (69).

The most evidence was observed for determinants of being active. Eleven MoAs were positively associated with being active across ten studies (55, 58, 61, 63, 67–69, 74, 78, 82). The most commonly reported MoAs were beliefs about capabilities (nine positive associations) and beliefs about consequences (six positive associations). Other commonly reported positive determinants of being active were emotion (69, 86), intentions (69, 78), and self-image (61, 68). Positive associations with being active were also observed in relation to reinforcement and goals (67, 69), subjective norms (78), values (68), and perceived susceptibility/vulnerability (61). A negative association between motivation and attitudes toward the behaviour and being active was reported in one study (63). Additionally a large UK study of people with mixed SMI reported no significant association between attitudes toward the behaviour and being active and (68). There was similarly no evidence that social/professional role and identity was a significant determinant of being active (69).

Two cross-sectional studies reported positive associations between emotion and reducing risks associated with smoking and diabetic foot problems (69, 71), and one prospective cohort study reported positive associations between emotion and reducing risk of smoking (81). Goals were negatively associated with reducing risks in one study (69). Cross-sectional data from one study showed no significant association between reinforcement, beliefs about consequences, intention, optimism, and social/professional role identity and reducing risks behaviours (69). Additionally longitudinal data from one prospective cohort study showed no significant association between motivation or self-image and reducing risks (87).

No positive associations were reported for links between determinants of taking medications. All observations were drawn from one cross-sectional study (69). Goals were negatively associated with taking diabetes medication. No significant associations were reported for reinforcement, beliefs about consequence, intentions, optimism, and social/professional role identity and taking medications.

There was no evidence found for links between AADE-7 self-management behaviours and these MoAs: norms, needs, social learning/imitation, feedback processes, and general attitudes/beliefs.

#### Summary of Findings for Motivation

The certainty of evidence for associations between MoAs and AADE-7 health behaviours within the Motivation domain was generally rated as moderate, but some evidence for positive associations was drawn from studies with low and very low ratings. The bulk of the evidence about determinants of behaviours was captured within this domain, with 44 links between four behaviours (healthy eating; being active; reducing risks; monitoring) and 11 MoAs being reported as positive. Results for being active and healthy eating clustered around beliefs about capabilities and beliefs about consequences. Goals and intentions were also linked three times with these behaviours. There was less inconclusive evidence within this domain with only four studies reporting no associations across four behaviours. Negative associations were reported for motivation (being active), goals (taking medication; monitoring; reducing risks, and attitude toward the behaviour (being active), but these findings were reported in just two studies.

## Discussion

Using the novel MoA framework that comprehensively captures processes known to be associated with behaviour change, this review aimed to identify the determinants of self-management behaviours that underpin physical health in adults with SMI. The bulk of the evidence for associations between MoAs and self-management behaviours clustered around the super-ordinate Motivation domain in the COM-B framework. This finding lends further empirical support to the proposition that Motivation (which includes reflective and automatic processes) sits at the centre of the COM-B model and mediates behaviour via Capability and Opportunity (88). In keeping with the expert consensus exercise that mapped MoAs with behaviour change techniques (39), our review showed that being active mostly operated through beliefs about capabilities and beliefs about consequences. This finding is also consistent with evidence that the COM-B constructs of psychological capability and reflective motivation predict moderate-to-vigorous physical activity in healthy adults (88). Reflective motivation also underscores intentions, self-image, and perceived risk or perceived susceptibility which were also shown to be positively associated with being active in people with SMI alone and people with SMI and diabetes.

Outside of the capability and motivation constructs we also showed that environmental context and resources were important determinants of being active. Previous reviews have shown that lower self-efficacy and social isolation are correlated with lower physical activity participation in people with schizophrenia (89) and bipolar disorder (90). We found that physical activity in people with SMI is reported to be more frequent in those who are employed than unemployed. Social support from friends might also be important in promoting engagement with physical activity in people with SMI. However, access to employment and social support is likely to be closely linked with an individual's experience of SMI, as people who are experiencing low mood or acute psychosis (91), and who do not adequately respond to treatment (92), are less likely to access social support and employment (93).

There was less evidence and less consistency across available evidence about determinants of other self-management behaviours. Ten MoAs were linked with healthy eating, but none were reported more than once. It is worth noting that memory, attention, and decision processes, and behavioural regulation were negatively associated with healthy eating in people with SMI and diabetes. Both these MoAs include higher level cognitive processing that some people with SMI might find challenging. Cognitive deficits associated with attention and working memory are considered a central feature of schizophrenia (94). These deficits can make it difficult for people with schizophrenia to encode and arrange information, making self-evaluative tasks that require attention to multiple streams and sources of information and feedback difficult.

Reducing risks, monitoring, and taking medication are critical behaviours to self-managing long-term conditions, and this is especially the case in the context of SMI. We identified four studies of self-management in people with diabetes and SMI. Lower diabetes related distress was associated with less smoking and more frequent blood glucose monitoring among people with SMI and diabetes. People with SMI are three times as likely as the general population to smoke (95) and are more likely to become nicotine dependent and develop smoking related illnesses (96). Furthermore, people with SMI commonly hold the belief that smoking relieves their depression and anxiety (97). There is good evidence that bespoke smoking cessation interventions that include behavioural support from mental health practitioners and pharmacological therapies can help people with SMI to quit smoking and that such approaches are not detrimental to mental health (98). The role of emotion in determining engagement with self-management behaviours suggests that managing mood, and symptoms of SMI, could be important to successful behaviour change such as reducing risks of smoking. The prevalence of depression is about 40% in people with schizophrenia and is known to negatively affect quality of life, psychosocial functioning, and medication adherence (99). A meta-analysis by Firth et al. of motivators and barriers to physical activity in SMI reported the most important motivators were losing weight, improving mood and reducing stress. It found barriers were related to mental health symptoms such as depression and stress (100). The ability to self-manage emotional well-being is captured by the healthy coping behaviour in the AADE-7 framework. However, we did not identify any studies that measured associations between determinants and healthy coping, possibly because our focus was mainly on determinants of physical health behaviours. However, healthy coping may be an important determinant of physical health behaviours, as the revised version of the AADE-7 framework places healthy coping at the centre of this self-management model on the basis that a positive attitude toward diabetes and self-management is critical to the mastery of the other six behaviours (48). Additionally, qualitative research has highlighted that people with SMI use unhealthy coping strategies, such as smoking (101), eating unhealthy foods (102), drinking alcohol and using illicit substances (103), to cope with the symptoms of mental illness (21) Going forwards, there is scope to better understand how the promotion of healthy coping in people with SMI might underpin successful engagement with other AADE-7 self-management behaviours associated with good health outcomes.

We also did not identify much evidence about what determines medication taking, but there was some signal that environmental context in the form of access to health services might play an important role in driving this behaviour among people with SMI and diabetes. Adherence to oral hypoglycaemic medication is known to be higher among adults with diabetes who take three or more other medications and have more frequent physical health checks (104). In the UK, primary care has been incentivised to offer physical health checks to people with SMI with the potential to improve the quality of care (105). However, in recent years many of these physical health indicators have been removed and the impact of this on people with SMI is unknown (106). Further research about which MoAs are likely to support people with SMI and long-term conditions to engage with health services is warranted. Here, the perspectives of health professionals and carers might be insightful as there may be some mechanisms that people with SMI are less likely to self-report (107). Furthermore, by focusing on evidence originating from people with SMI alone, the relationship between self-management and the organisation and delivery of care may be overlooked (87).

Despite there being good evidence for how health behaviour models might explain physical activity in the general population, few behaviour change interventions used in people with SMI are underpinned by theory. Only nine out of 32 studies included in a systematic review of behaviour change interventions to promote physical activity in people with SMI were based on theory (11). Social cognitive theory and self-determination theory accounted for over half of those studies that did use theory. Furthermore, only three of the 11 studies that reported positive outcomes for physical activity used theory, drawing on social cognitive theory, and in particular self-efficacy theory, and acceptance and commitment theory. Self-efficacy and intention of physical activity have previously been shown to be a determinant of physical activity in people with SMI and depression (108), but in people with schizophrenia alone there is no evidence that trans-theoretical model mediators of change, such as exercise self-efficacy, are predicative of physical activity (109). Moreover, the majority of intervention studies that aim to increase physical activity in people with SMI have failed to target motivation for physical activity. Studies that have attempted to incorporate motivational techniques within interventions have observed no change in physical activity in people with SMI, pointing to the need for the systematic appraisal of the theoretical determinants of motivation for behaviour change in people with SMI (110).

### Strengths and Limitations

A major strength of this systematic review is that it has operationalised the novel MoA framework using the COM-B model, possibly for the first time, to synthesise published evidence about links between determinants of self-management behaviours in people with SMI, including people with SMI and diabetes. The strengths of the MoA framework stem from the fact that it is based on the result of evidence synthesis and expert consensus, with a series of subsequent triangulation studies that sought to integrate the previous findings quantitatively and through expert consensus. This approach has resulted in evidence that builds upon the previous BCT Taxonomy V1 (36) and provides evidence of 1,456 BCT–MoA links, with the nature of the link (link/non-link/inconclusive) and type of evidence available in a heatmap format in the web-based Theory and Technique Tool (41). This work is part of a broader effort toward an ontology of human behaviour change (111–113), that is extending into intervention source and intervention mode of delivery (114). As such, the MoA Framework explicitly offers the benefits of an ontological approach that links intervention content to behaviour change techniques. This information is key for both systematic reviewers and intervention developers seeking to inform and or evaluate intervention development (43). Our review identified evidence of associations between AADE-7 health behaviours and all 14 domains of the TDF, but we also found evidence of associations for seven additional determinants that have been proposed as part of the MoA framework. This expands the scope of mapping MoAs to BCTs to change target behaviours and affect clinical outcomes, thus supporting the utility of this extended framework over and above the TDF alone.

We did not find studies of people with other long-term conditions so we cannot be sure this evidence extends beyond diabetes. Methodological strengths of the review include independent assessment of study eligibility by two reviewers and checking of data extraction and quality appraisal by a second reviewer. We conducted comprehensive searches but it is possible some evidence was missed through exclusion of conference abstracts and dissertations and a top-up search was conducted in only two databases.

We did not rate quality of included studies based on sample size. Nor did we exclude studies that reported smaller sample sizes. There is little empirical evidence that sample size drives risk of bias and smaller studies can still make a significant contribution to innovative and translational research (115). Our review aimed to identify all relevant evidence about the association between MoAs and physical health self-management behaviours, including from smaller studies. This approach offered greater opportunities to synthesise findings across multiple studies and minimised reporting bias about how associations between determinants and self-management behaviours clustered around particular MoAs. Additionally, excluding studies with smaller samples might lose information about important sub-groups, including those with SMI and diabetes.

Using the MoA framework can make it difficult to maintain specificity in how determinants are identified, especially among those existing within broader domains such as environmental context and resources, with implications for the appropriateness of subsequent selection of behaviour change techniques. More descriptive and nuanced data about whether determinants acted as barriers or motivators could also have been lost through the methods used, pointing to the need for qualitative studies about drivers of self-management behaviours in people with SMI. Additionally, the framework does not describe relationships between mechanisms established by social cognition models of behaviour and behaviour change (116). Hypothesised links, such as between perceptions, cues and intentions, can be overlooked by amalgamating theories into an integrative framework. However, MoAs are accessible to an interdisciplinary audience and can be organised using the COM-B system to aid understanding of their similarities and differences (33). Nevertheless, use of the MoAs in conjunction with the COM-B system creates overlaps (such as motivation within motivation) and two MoAs (Social Learning/Imitation and Behavioural Cueing) belong to more than one of the three overarching COM-B domains. The new MoAs are less clearly specified and inclusive than MoAs that originated in the TDF, which might account for why the majority of evidence fell within domains that were previously captured by the TDF. Further specification of the MoAs, particularly those that were not previously described in the TDF and which have clear links to social cognition models would make coding and application of evidence arising from evidence synthesis more accessible and feasible. An additional limitation of the MoA framework is that it does not account for how symptoms and health status can impact on a person's ability to engage in behaviour change. Our review is therefore unable to discern how psychiatric symptoms might affect engagement in behaviour change interventions in people with SMI.

We assessed the strength and direction of associations using conventional measures of statistical significance for univariate analyses that assessed the relationship between MoAs and self-management behaviours. This approach might limit the robustness of the synthesis as tests of significance and non-significance are dependent on a range of factors such as sample size or quality which is not captured using these methods. Furthermore, many of the included studies had small sample sizes that precluded the use of multivariate regression analyses that would offer adjusted and more accurate assessments of predictors of self-management behaviours.

A further limitation of the review is that it included mainly moderate to low quality cross-sectional evidence that cannot attribute causality, but this was partly a result of excluding intervention studies to avoid including associations between determinants and behaviours that had been modified by behaviour change techniques. This approach effectively restricted the evidence base to single group cohort designs. Most of the included studies did not use valid measures of behaviour. This was especially true for physical activity. Only one study used an accelerometer to objectively measure activity but the small sample size precludes drawing firm conclusions. There were also studies which compared SMI groups to non-SMI control groups and only the SMI group data could be extracted from these studies. It was not always straightforward to extract and map behavioural determinants but mapping to MoAs was performed by researchers with past experience of applying the framework to evidence of behavioural determinants in SMI groups. We enhanced the internal validity of this process by consulting linked evidence, such as qualitative studies, to help pinpoint the most appropriate MoA, with a second reviewer checking all allocation of evidence to MoAs.

### Implications for Research and Intervention Development

Our heat-map matrix of MoAs and self-management behaviours, organised under the broader COM-B model of behaviour change, allows for easier identification and possible adaptation of candidate behaviour change techniques using existing resources such as the online theory and techniques tool (117). Using this tool, we can map MoAs that appear to be important determinants of health behaviours in people with SMI to BCTs with evidential links, thereby informing the next phase of work to develop interventions to support self-management of physical health in people with SMI. By way of example, beliefs about consequences, beliefs about capabilities, environmental resources and context, emotion, intention, and motivation were MoAs that were most commonly associated with AADE-7 health behaviours. Based on the theory and techniques tool these six MoAs are linked with 28 BCTs that are organised under 12 super ordinate categories in the Behaviour Change Taxonomy v1 (36). Four of these BCTs are linked to more than one MoA suggesting that there are opportunities for targeting multiple MoAs with single BCTs.

Feedback and monitoring are among the BCTs linked to the MoAs that we identified as being associated with health behaviours under the Motivation domain of the COM-B. This finding tallies with the results of the STEPWISE process evaluation which showed that participants who wanted to lose weight wanted closer monitoring and healthcare professionals wanted to monitor weight outcomes too, but such a focus on monitoring lay outside the scope of the intervention. Going forwards, the advent of wearable technology to objectively measure physical activity, behaviour change applications for smartphones, and devices that allow continuous glucose monitoring are likely to transform the capacity of behaviour change interventions to facilitate monitoring. There is evidence that the use of wearable technology and digital applications are acceptable among adults who take part in facilitated and remotely delivered behaviour change interventions to reduce the risk of long-term conditions (118). The relevance of such approaches is, however, untested in people with SMI and future trials are needed to assess feasibility and acceptability of the use of such technology in the delivery and assessment of behaviour change interventions in these populations.

## Conclusion

This review provides an evidential basis for the development of appropriate and theory-based behaviour change interventions for managing physical health in adults with SMI. We synthesised evidence about 21 determinants of physical health self-management behaviours known to be important in people with SMI and people with SMI and a long-term condition. Organisation of evidence within the MoA framework facilitates the identification of behaviour change techniques with hypothesised links to determinants. Many of these determinants overlap and stem from reflective and automatic motivational processes. Critical determinants for being active and healthy eating were beliefs about capabilities and beliefs about consequences. There was less evidence about what determines other self-management behaviours but emotion and environmental context and resources appear to be important determinants of reducing risks and taking medications. The next phase of research and development should involve drawing up a shortlist of candidate BCTs and the involvement of healthcare professionals and people with lived experience of SMI to support decisions about how they are delivered using methods such as expert consensus and co-design.

## Data Availability Statement

The original contributions presented in the study are included in the article/Supplementary Material, further inquiries can be directed to the corresponding author/s.

## Author Contributions

NS, JT, PC, EP, IK, CK, SA, JW, and JL wrote the protocol. CK, JT, PC, SA, EP, JL, AB-K, BY, JBr, CC, and IK screened titles and abstracts. BY, AB-K, JBr, and CC screened full text records. CC, JBr, PC, AB-K, BY, and JT assessed quality of included studies. PC wrote the first complete draft of the manuscript. All authors edited the manuscript for substantive intellectual content.

## Author Disclaimer

The views and opinions expressed therein are those of the authors and do not necessarily reflect those of the NHS, NIHR, or the Department of Health and Social Care. URKI does not necessarily endorse the views expressed by the authors.

## Conflict of Interest

The authors declare that the research was conducted in the absence of any commercial or financial relationships that could be construed as a potential conflict of interest.

## Publisher's Note

All claims expressed in this article are solely those of the authors and do not necessarily represent those of their affiliated organizations, or those of the publisher, the editors and the reviewers. Any product that may be evaluated in this article, or claim that may be made by its manufacturer, is not guaranteed or endorsed by the publisher.
